# Eradication of proliferative verrucous leukoplakia with toll-like receptor 7 agonist (topical imiquimod): a case report

**DOI:** 10.3389/fonc.2024.1473889

**Published:** 2024-11-15

**Authors:** Melanie Townsend, Alexandra E. Kejner, Farzad Nourollah-Zadeh, Fabio A.P. Rizzante, Tina R. Woods, Sonali Rathore, Douglas Alterman, Sorin Teich, William G. Albergotti, Jason G. Newman, Angela J. Yoon

**Affiliations:** ^1^ Department of Otolaryngology Head and Neck Surgery and Communicative Disorders, University of Louisville, Louisville, KY, United States; ^2^ Department of Otolaryngology-Head & Neck Surgery, Medical University of South Carolina, Charleston, SC, United States; ^3^ Department of Biomedical & Community Health Sciences, Medical University of South Carolina, Charleston, SC, United States; ^4^ Department of Reconstructive & Rehabilitation Sciences, Medical University of South Carolina, Charleston, SC, United States

**Keywords:** Proliferative verrucous leukoplakia, topical imiquimod, toll-like receptor 7 agonist, oral neoplasm, treatment

## Abstract

Proliferative verrucous leukoplakia (PVL) is an aggressive and distinct type of oral precancerous lesion characterized by warty surfaced white plaque diffusely involving oral mucosa. Surgical excision is the treatment of choice. However, PVL has persistent and recurrent growth patterns, requiring multiple surgical procedures. Surgical intervention is especially challenging if PVL extends between teeth limiting access. These interproximally located lesions have a high propensity to undergo malignant transformation. We report a case of a 53-year-old man with recurrent PVL diffusely covering the maxillary and mandibular gingiva. Despite complete surgical excisions, PVL recurred, and a focal area in the interproximal mandibular gingiva progressed to invasive squamous cell carcinoma requiring marginal resection. The remaining PVL areas were treated with topical imiquimod (toll-like receptor 7 agonist) for six months, resulting in complete clinical and histological resolution. Topical agents can cover a larger surface area and penetrate in between interproximal areas. Importantly, it allows for maximal local exposure with minimal systemic toxicity, essential for long-term treatment and prophylactic use of the agent to prevent relapse.

## Introduction

Proliferative verrucous leukoplakia (PVL) is a long-evolving, aggressive, and irreversible precancerous condition with a malignant transformation rate of 63.9% and a recurrence rate of 67.2% (1–3). Etiology is unknown, with a weaker association with tobacco and alcohol consumption (3). Although the PVL pathogenesis seems to involve the activation of immunological pathways that can be associated with human papillomavirus (HPV) infection, there is no firm evidence of HPV involvement (4). The most common site is gingiva, in which lesions initially appear as white patches that proliferate with time to become multifocal, later confluent, and diffuse verrucous plaques (1). Current treatment modalities include complete removal with scalpel excision or laser ablation (1–3). Despite research efforts, no approved therapeutic strategies to halt PVL recurrence exist (1). Toll-like receptor (TLR) 7 agonists, imiquimod, FDA-approved medication for cutaneous neoplastic lesions, have shown promise in off-label use for other oral precancerous and cancerous lesions (5, 6). There are limited reports of successful treatment of PVL with imiquimod. Here, we report a case of the off-label use of imiquimod to treat recurrent PVL and prevent relapse.

## Narrative

**Figure 1:**
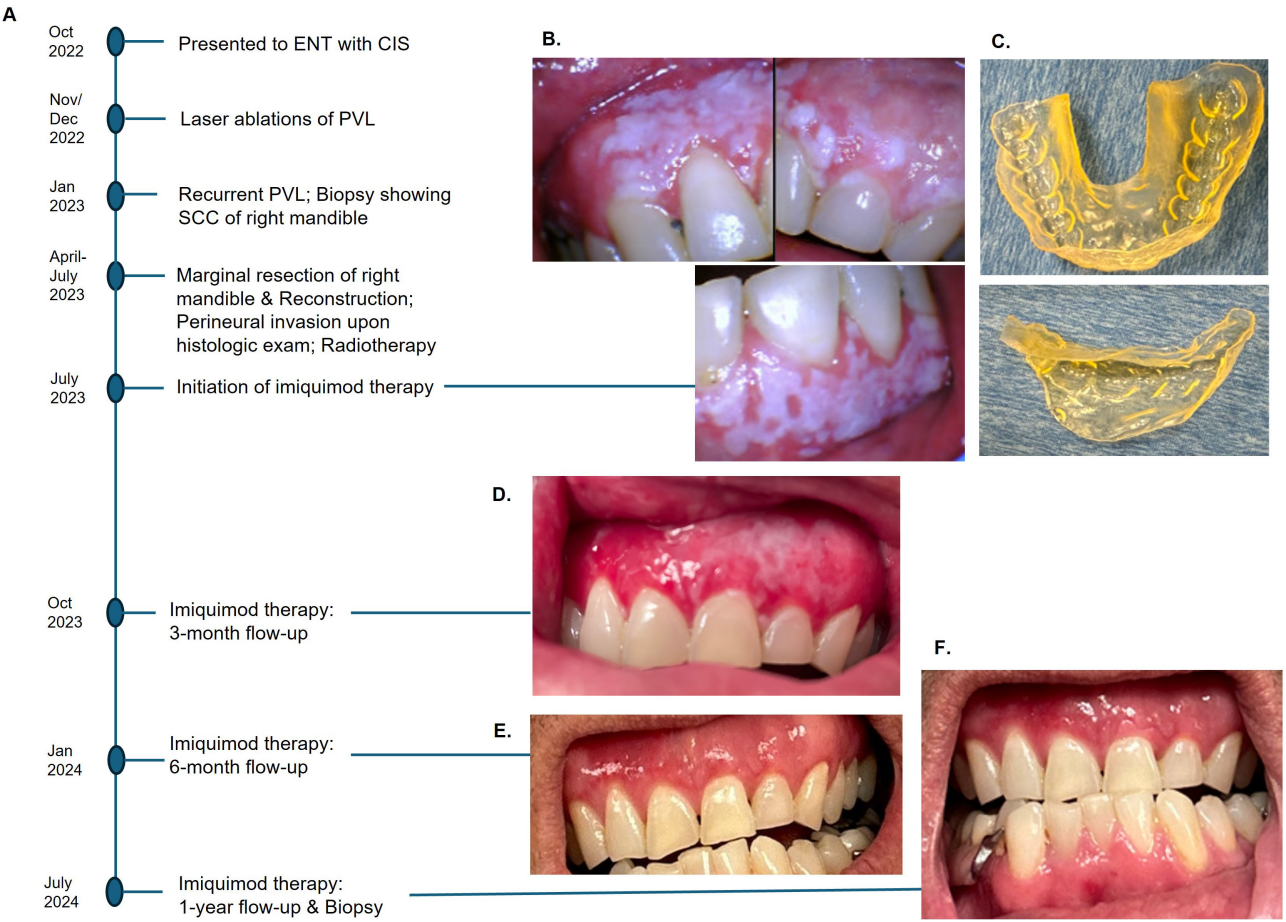
Events in chronological order **(A)**. Diffuse PVL of maxillary gingiva and left mandibular gingiva after marginal resection of the right posterior mandible **(B)**, 3-D printed customized tray to hold imiquimod cream **(C)**. A significant reduction of PVL was observed at 3-month follow-up **(D)**, and no clinically visible lesion at 6-month post-therapy **(E)**. No clinical or histologic evidence of recurrence after a year with continued use of imiquimod **(F)**.

A 53-year-old man presented to the Otolaryngology in 2022 with histology-confirmed carcinoma-*in-situ* of the right maxillary gingiva presenting as flat, ~3 cm erythroleukoplakia and a smaller lesion of mandibular gingiva. He had no other illnesses or history of smoking or alcohol abuse. Additional biopsies were obtained to assess for invasive squamous cell carcinoma (SCC), which showed high-grade epithelial dysplasia but no carcinoma. The *in-situ* hybridization for high-risk human papillomavirus types 16 and 18 was negative. The patient had KTP laser ablation of all the maxillary and mandibular lesions. At a 1-month follow-up, the lesion recurred in both maxillary and mandibular gingiva, and re-biopsy showed moderate epithelial dysplasia. A second round of laser ablation was performed. At the next 1-month follow-up, recurrent PVL diffusely involving maxillary and mandibular gingiva was noted. Repeated biopsies showed moderate epithelial dysplasia in the maxillary gingiva but invasive SCC of the right posterior mandibular gingiva. PET/CT showed no evidence of nodal involvement or distant metastasis (T1N0M0). Marginal resection of the posterior mandible along with the extraction of three teeth and the sentinel lymph node biopsy of the neck were performed. The patient also underwent oral cavity brachytherapy 3000 cGy in 10 fractions in 1 week (2 fractions per day). The timeline is summarized in **Figure 1A**.

The patient was referred to an oral and maxillofacial pathologist for non-surgical management of residual PVL. At baseline, an intraoral camera was used to capture all lesions in detail (**Figure 1B**), and cone-beam CT was performed to ensure no osseous involvement. A 3-D printed custom tray was fabricated to allow retention of the imiquimod 5% cream in place (**Figure 1C**). The patient was instructed to apply two single packets of imiquimod cream on the affected mucosal areas, one for maxillary gingiva and one for mandibular gingiva, once every other day before bedtime. The patient was directed to massage the cream into mucosa using gloved fingers, place the custom tray over it, and squeeze the appliance to cause the cream to diffuse between the teeth to reach interproximal areas. After 20 minutes of application, the cream was rinsed thoroughly with water.

**Figure 2:**
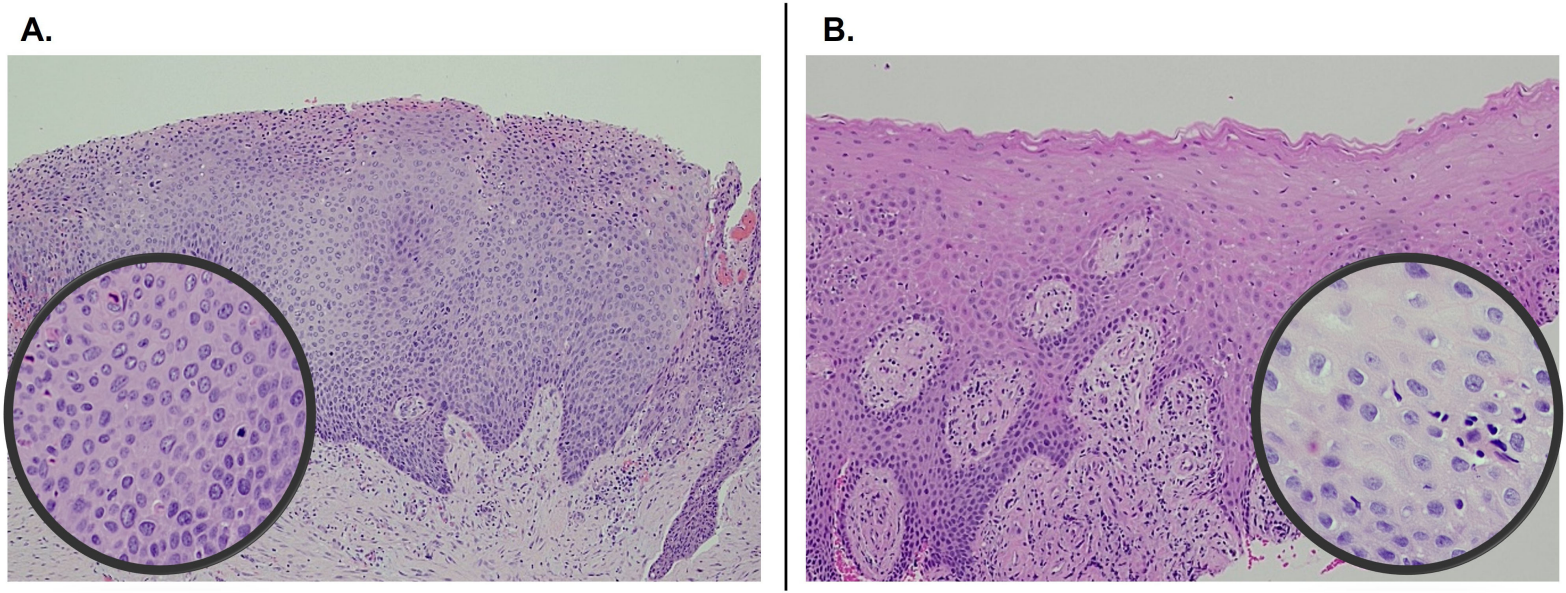
Pre- and post-treatment comparison of histopathology of right maxillary gingiva showing carcinoma-*in-situ* at initial presentation **(A)** and normal mucosa 1-year following imiquimod treatment **(B)**. Both photomicrographs were captured at 100x and the insets at 400x.

A significant resolution of PVL was noted at the three-month post-therapy visit, with thinner leukoplakia foci remaining in the left maxillary gingiva (**Figure 1D**). No significant adverse effects were reported. The entire gingiva had an erythematous appearance, most likely due to application site reaction. By six months post-therapy, the lesion was no longer visible (**Figure 1E**). The patient opted to continue with two packets every other day to prevent recurrence. At 12 months follow-up, there was no evidence of PVL recurrence (**Figure 1F**). Multiple biopsies were taken from the maxillary and mandibular gingiva, which all showed normal mucosa without evidence of epithelial dysplasia. Photomicrographs comparing the initial biopsy of the right maxillary gingiva showing carcinoma-*in-situ* and the post-treatment biopsy of the same location demonstrating normal mucosa are illustrated in **Figure 2**. The patient will continue with the twice-a-week imiquimod regimen for a year. If no recurrence is observed, the dose schedule will be changed to once a week to prevent recurrent PVL.

## Discussion

Proliferative verrucous leukoplakia (PVL) is difficult to manage due to the widespread nature of the lesion requiring extensive surgical excision (1–3). The challenge is further compounded by the lesions affecting hard-to-reach interproximal dentate areas, a high recurrence rate, and malignant transformation potential; this, in turn, necessitates multiple surgeries and frequent biopsies (1–3). The most commonly used treatment modalities for PVL are scalpel excision, laser ablation, and electrocautery, among other invasive procedures (1). In attempts to prevent recurrence, various therapies, including retinoids, vitamin A, antioxidants, and cyclooxygenase inhibitors, have been tried without clear efficacy (3).

Imiquimod is a toll-like receptor (TLR) 7 agonist exhibiting anti-tumoral effects mediated by initiating apoptosis of neoplastic cells and activating innate and adaptive immunity (7, 8). Imiquimod increases tumor cell apoptosis by shifting the pro- and anti-apoptotic Bcl factors toward the pro-apoptotic Bax protein and by stimulating the release of mitochondrial cytochrome c into the cytosol, activating caspase-9 and caspase-3 (9, 10). Upon binding to TLRs, imiquimod activates nuclear factor-kB, which stimulates the production of pro-inflammatory cytokines (interleukin-6/8) and other components of innate immunity (8). The end result is T cell-mediated anti-tumoral response (8–11). The time for absorption of drugs into the oral mucosa is short, and any residual medication is washed with saliva after 5 to 10 minutes of application (12, 13). Residual imiquimod mixed in saliva can irritate the oropharynx, resulting in pharyngitis. With the trays allowing retention of the imiquimod in place, the medication was applied for 20 minutes and then thoroughly rinsed.

There are a limited number of studies assessing the efficacy of imiquimod in managing PVL (**Table 1**). Martinez-Lopez et al. (14) reported successful treatment of a 66-year-old woman with PVL of the lower lip and gingiva, applied three nights a week for six weeks, and no recurrence was observed at the six-month follow-up. Alhadlaq et al. (15) reported the study of oral leukoplakia treatment with imiquimod, in which 70.6% of the lesions were multi-focal/proliferative. Imiquimod was applied five days a week for six weeks. At six months follow-up, 75% of the patients were recurrence-free. There are few other reports of utilizing imiquimod to treat multifocal, diffuse, or recurrent oral neoplasm, including oral precancer and cancer and florid oral papillomatosis (16–20).

**Table 1:** Reports and studies of imiquimod use for oral neoplasm.

Study/Case report (Reference number)	Type of lesion	Dose schedule	Complete response	Duration of response
Case report (14)	PVL	3x per week for 6 weeks	100%	6 months
Study (15)	Oral precancer including PVL	5x per week for 6 weeks	31.6%	6 months in 75% of patients
Study (16)	Oral precancer	3x per week for 6 weeks	6.66% with +2-unit change	6 months
Case report (17)	Oral precancer	Daily for 6 weeks	100%	18 months
Case report (18)	Recurrent oral cancer	Daily for 2 weeks, tapered to once weekly after 2 months	100%	Continued once weekly for 4 years without recurrence
Case report (19)	Florid oral papillomatosis	Four cycles of 3x per week and 2 weeks pause	0%	Not available
Case report (20)	Florid oral papillomatosis	Every other day for 16 weeks	100%	2 years

The adverse events associated with imiquimod include application site mucositis and discomfort, nausea, and influenza-like symptoms (11). The serum level of active drugs is very low or undetectable during the imiquimod therapy (21). While the exact cause of systemic adverse events is unclear, studies show that imiquimod-related flu-like symptom is encountered during the first treatment cycle in patients treated on the head and neck compared to the body and are independent of age and circulating levels of cytokines during treatment (21). A long-term application of imiquimod for a mean duration of 6.3 years demonstrated safety without serious adverse events (22). Similar to our case, other studies reported that imiquimod is effective and safe for patients with oral precancers. Importantly, the studies that had patients placed on a long-term maintenance dose of imiquimod report no recurrence (17, 18, 20).

Metronomic chemotherapy (MCT) and drug repurposing (i.e., off-label use of imiquimod) have emerged as promising therapeutic approaches for the treatment of various neoplasms (23). Similar to imiquimod, MCT allows oral administration of chemotherapeutic regimens at lower doses without long drug-free intervals and exerts an immunomodulatory effect in the tumor microenvironment (23). However, MCT is associated with more severe systemic toxicity compared to imiquimod, which includes nausea, minor-grade fatigue, mild to severe leucopenia, lymphopenia, neutropenia, and anemia (11, 23).

Our follow-up period is limited to six months after the patient had a complete resolution of PVL. With a mean follow-up of 79 months, the recurrence rate was 67.2% [95% CI: 48.3-81.8] (2). The average time to malignant transformation is four years (24). Hence, a long-term follow-up is needed to accurately assess the efficacy of imiquimod in preventing recurrence and malignant transformation.

## Conclusion

We report the efficacy and safety of imiquimod therapy for an aggressive precancerous oral lesion with high malignant transformation potential. PVL is also associated with a high recurrence rate. As such, long-term maintenance therapy after the initial resolution of the lesion may be necessary to prevent relapse.

## Data Availability

The original contributions presented in the study are included in the article/supplementary material. Further inquiries can be directed to the corresponding author.
